# Small cell carcinoma of the urinary tract: a case report

**DOI:** 10.4076/1757-1626-2-7743

**Published:** 2009-07-24

**Authors:** Diomidis Kozyrakis, Panteleimon Papadaniil, Stefanos Stefanakis, Efstathios Pantazis, Alkiviadis Grigorakis, Konstantina Petraki, Dimitrios Malovrouvas

**Affiliations:** 1 Department of Urology, “Evagelismos” General Hospital of AthensIpsilandou 45-47, 10676, AthensGreece; 2 Department of Uropathology, “Evagelismos” General Hospital of AthensIpsilandou 45-47, 10676, AthensGreece

## Abstract

Neuroendocrine small cell carcinoma of the urinary tract is rarely encountered and very few cases have been reported in the literature. Herein we describe a case of small cell malignancy located contemporarily in the ureter and the bladder.

## Introduction

Neuroendocrine small cell carcinoma (SCC) is usually diagnosed in the lung but its extrapulmonary counterpart is rarely encountered. The extrapulmonary SCC has been identified in the gastrointestinal tract, the ear, nose and throat region, the cervix, the genitourinary tract in men, the upper respiratory system, the lymphatics, the thymus and the peritoneum [1,2]. Among the organs of the genitourinary tract, the most common primary site of the disease is the bladder, followed by the prostate, the kidney and the upper urinary tract [1,3].

Small cell carcinoma of the urinary tract is a rare disease. Herein, we describe one case with SCC located contemporarily in the ureter and the bladder.

## Case presentation

A 78-year-old Greek male Caucasian presented to the emergency department due to gross painless intermittent hematuria. One month before presentation, he observed a similar symptom but it resolved spontaneously within 24 hours and the patient did not ask for medical consultation. He used to be a heavy smoker for over 33 years. He suffered from arterial hypertension, diabetes mellitus type II and prostate hyperplasia and he was under treatment with lisinopril dihydrate (20 mg once a day), glimepiride (2 mg twice a day) and tamsulosine hydrochloride (0.4 mg per day) respectively. A family history of neither painless hematuria nor urothelial malignancy has been referred.

**Figure 1.**
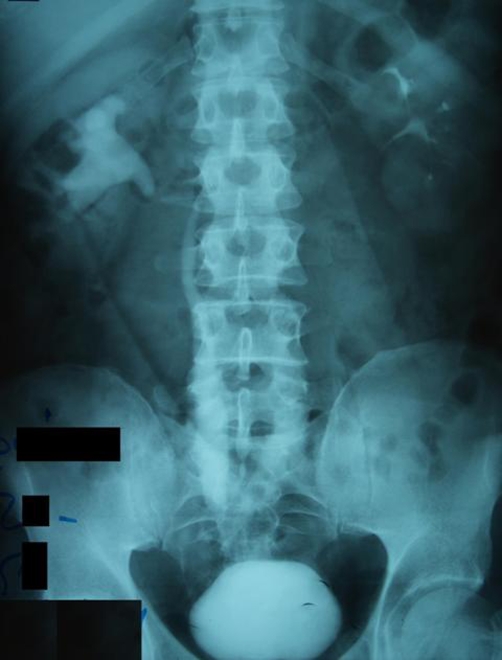
Preoperative IVU showing a filling defect at the lower one third of the right ureter, proximal hydroureter, hydronephrosis and ureteral kinking.

**Figure 2.**
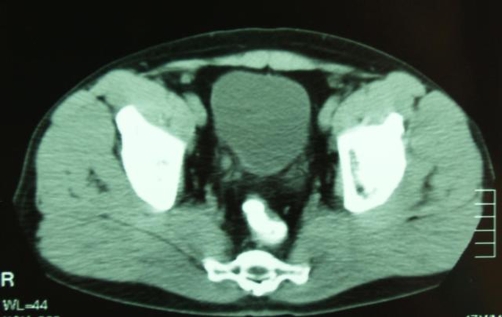
Preoperative CT imaging showing a lesion of the lower one third of the right ureter in close proximity to the bladder wall.

The physical examination was unremarkable. The intravenous urography (IVU) showed a 2 cm-long lesion at the lower one third of the right ureter (Figure 1). The cystoscopy identified two tumor sites in the bladder. In addition, the urine jets coming from the right ureteral orifice were admixed with blood. Urine cytology was positive for high grade urothelial cancer. The abdominal CT imaging confirmed the presence of a ureteral tumor (Figure 2). Both the abdominal and the chest CT imaging revealed no signs of distant metastases or lymphatic involvement.

**Figure 3.**
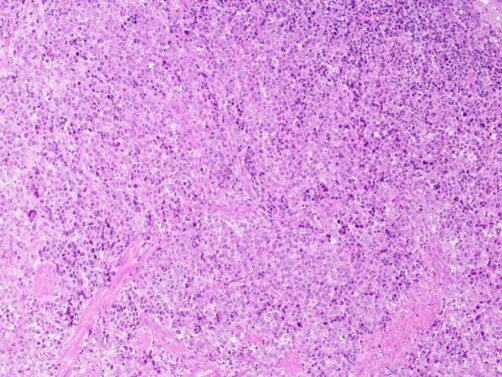
Pathologic examination of the surgical specimen. Small cell component of the carcinoma on light microscopy with H & E staining (×200).

**Figure 4.**
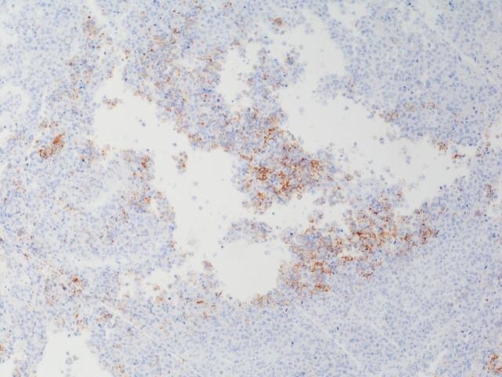
Immunohistochemical staining of the surgical specimen. Cytoplasmic expression of CD-56 in the SCC component (×200).

Transurethral resection of the bladder tumors (Tur-BT) was initially performed. The postoperative recovery was uncomplicated and a few days later the patient underwent right nephroureterectomy with removal of the ipsilateral bladder cuff. The surgical margins were free of disease. Macroscopically, a 1.7 cm long, oval-shaped and yellow-to-pale colored lesion was identified in the ureteral specimen. The histological examination revealed a neuroendocrine small cell carcinoma admixed with a high grade urothelial carcinoma and with foci of squamous cell differentiation (Figure 3). In situ carcinoma was also diagnosed. The lesion extended beyond the ureteral wall, infiltrating the periureteral fat. The immunohistochemical staining of the specimen was positive for CD56, synaptophysin (Syn) and chromogranin (Chr), confirming the neuroendocrine origin of the tumor (Figure 4). Both the neuroendorine and the urothelial component of the carcinoma expressed a 70% positive staining for Ki-67/MiBi.

The renal parenchyma, the renal pelvis and the upper and mid ureter were free of disease. The bladder tumors had similar histological features to those of the ureter but none of them was infiltrating the muscular layer of the bladder wall.

Considering the aforementioned clinical and pathological findings and despite the negative results of the preoperative staging, a thorough investigation of the respiratory system was performed in order to exclude the lung from being the primary site of the malignancy. The high resolution spiral CT, the bronchoscopy examination, and the cytology of the sputum, the bronchoalveolar lavage and the brush specimen were all negative for pulmonary malignancy.

Postoperatively, renal dysfunction ensued (serum creatinine 2.2 mg/dl) and adjuvant chemotherapy (CMT) was not administered. Within 6 months, distant metastasis developed in the lung and shortly afterwards the patient died.

## Discussion

As far as we know, only 18 other cases of small cell carcinoma of the ureter and pelvis (SCC-UP) have been published in the literature [4-6]. A large series of 10 cases with SCC of the urinary system has been previously presented by Chuang & Liao [7].

Risk factors for the disease have not been documented so far. It is of particular interest the fact that most patients come from the Far East region [4]. It is unknown weather environmental conditions, geographic and local factors or dietary habits predispose to this form of disease.

The role of the uropathologist is of utmost importance for a correct diagnosis to be established. Histologically, the SCC-UP is usually admixed with other malignancies, mainly transitional cell carcinoma (TCC) and rarely with squamous cell carcinoma, adenocarcinoma and sarcoma [5,8]. On light microscopy, small cell carcinoma is consisted of small, round or oval shaped cells, with a prominent nucleus, scant cytoplasm and granular chromatin. A high mitotic index may be observed [7,9,10]. In controversial cases, positive immunohistochemical staining for two or more of the neuroendocrine markers eg. CD-56, neuron specific enolase (NSE), Syn, Chg, might contribute to the correct diagnosis [9,10]. We confirmed positive staining for CD-56, which could also be implicated in novel targeted-molecular therapies. It has already been reported that C-kit, EGFR, BCL2 and CD-56 represent potential targets of molecular therapy and have been identified immunohistochemically in SCC of the prostate [10]. A detailed immunohistochemical analysis of the SCC-UP specimens, with a panel of antibodies against the aforementioned potential therapeutic targets, has not been performed so far.

Metastasis to the ureter, originating from a primary SCC of the lung, although rare, should be excluded during the diagnostic work up [2]. A lymphoma of the urinary tract and an undifferentiated urothelial carcinoma (uUC) could also be confused with the SCC-UP. Leukocyte common antigen (LCA) and CD44-v6 staining might contribute to the diagnosis of lymphoma and uUC respectively [9].

The vast majority of SCC-UP patients have at least a muscle invasive disease at presentation [4-7]. The patient we are presenting is not an exception, suffering from a muscle invasive SCC of the ureter and a non muscle invasive counterpart in the bladder. To our knowledge, no similar case has been presented in the past.

Surgery (nephroureterectomy or nephrectomy) is the primary treatment in the majority of SCC-UP patients but usually cannot achieve adequate control of the disease [4-7]. In the treatment of SCC of the bladder (SCC-B), cisplatin-based CMT has been frequently combined with surgery [2,3]. Mackey et al. [3] published that the platinum-based chemotherapy is the only statistical significant factor for improved survival in SCC-B patients and this finding should be considered in the treatment of SCC-UP. Radiation therapy alone has rarely been administered in SCC patients. It is usually combined with CMT, but the overall outcome is poor [2].

The lymphatics, the lung, the bones and the liver are common metastatic sites of the SCC-UP [6]. Generally speaking, in extrapulmonary SCC, the extent of the disease determines the survival and should be considered in determining the prognosis of SCC-UP patients [2]. Vimentin staining might also have a prognostic role. It has been published that the positive staining is correlated with high metastatic potential and reduced survival [7].

## Conclusion

Irrespectively of the treatment administered, SCC-UP patients face a dismal prognosis. The majority of them die within a year after the initial diagnosis [4,6,7]. Currently, debulking treatment associated with CMT could offer some survival benefit in selected patients [4]. In the future, targeted molecular therapy might radically change our therapeutic approach and improve survival of the patients with rare type of malignancy [10].

## Abbreviations

Chr: chromogranin
CMT: chemotherapy
CT: computed tomography
IVU: intravenous urography
LCA: leukocyte common antigen
NSE: neuron specific enolase
SCC: small cell carcinoma
SCC-B: SCC of the bladder
SCC-UP: small cell carcinoma of the ureter and pelvis
Syn: synaptophysin
TCC: transitional cell carcinoma
Tur-BT: transurethral resection of the bladder tumor
uUC: undifferentiated urothelial carcinoma
